# A Novel Macro-Level Model in Evaluating Health and Safety Training Based on Virtual Reality

**DOI:** 10.3390/ijerph22091378

**Published:** 2025-09-03

**Authors:** Antonella Pireddu, Claudia Giliberti, Alessandro Innocenti, Carla Simeoni, Michela Bonafede

**Affiliations:** 1 Technological Innovations and Safety of Plants, Products and Anthropic Settlements Department (DIT), Italian National Institute for Insurance Against Accidents at Work, INAIL, 00144 Rome, Italy; c.giliberti@inail.it (C.G.);; 2 Department of Social, Political and Cognitive Sciences (DISPOC), LabVR UNISI, University of Siena, 53100 Siena, Italy; 3 Occupational and Environmental Medicine, Epidemiology and Hygiene Department (DIMEILA), Italian National Institute for Insurance Against Accidents at Work, INAIL, 00144 Rome, Italy

**Keywords:** virtual reality, training life cycle, health and safety training management, VR training metric, virtual reality training evaluation model, impact, effectiveness

## Abstract

This document proposes a new evaluation model to be applied to a training course on health and safety at work based on virtual reality. The model refers to three macro-levels (design, delivery, and evaluation), which extend throughout the training life cycle. At macro level 1, design, the quality of the model intended for the virtual reality experience is evaluated, as well as its adaptation to the work environment and its compliance with applicable voluntary and mandatory standards; in macro level 2, delivery, the performance of the model, the individual reactions of users with headsets, their performance and psycho-physical state, the time, and the score achieved are evaluated; in macro level 3, evaluation, the long-term effects of subjective training and the social and economic impact that virtual reality training has had on the organisation are evaluated. The study investigates assessment models for virtual-reality-based occupational health and safety courses and identifies a model outlining general criteria that can be adapted to several types of courses and different work sectors. By examining the typical stages of the training life cycle and drawing on training evaluation models such as Kirkpatrick or Molenda and Information and Communication Technology metrics, the study identifies the key elements for assessing the effectiveness of virtual reality training in occupational health and safety.

## 1. Introduction

In recent years, virtual reality (VR) has emerged as an innovative tool capable of radically transforming training methods by providing immersive environments and highly realistic simulations. However, while VR presents significant advantages, it is not psychologically neutral and can generate both benefits and critical effects, especially when not tailored to the cognitive and emotional characteristics of the user. Training has become a regular feature of not only industrial and corporate settings but also of governmental and non-governmental organisations. Training has successfully been used to reduce errors in such high-risk settings as emergency rooms, aviation, and the military [1]. The main advantage is the effectiveness of VR as a tool for introducing contextual insights into work-related experiments. This element, which is difficult to achieve “in the field”, where the realism of the context is achieved at the detriment of control, replicability, and safety, mitigates the illusion of contextlessness of the standard laboratory approach. Furthermore, in VR, the perception of experimental subjects can be managed and evaluated through the sense of presence caused by immersion in the virtual environment. Therefore, it is not surprising that recent research emphasises the importance of post-experimental surveys and questionnaires that measure how VR engages the senses of participants [2]. According to Vigoroso et al., in the assessment of serious games, almost half of the studies included in their review evaluated games in terms of usability, playability, satisfaction, and users’ propensity to adopt VR as a new method of safety training [3]. The effectiveness of training becomes important in view of the time and resources committed to the training and development activity. Many countries have made considerable progress in digital transformation, improving their telecommunications infrastructure and online services and investing in human capital development. Worker health and safety training is also part of this transformation [4].

### 1.1. On the Technological Gap in the World

In discussing the report on science, technology, and innovation for sustainable development (Report of the Secretary-General, seventy-eighth session, Item 20 of the provisional agenda, Globalization and interdependence) (https://digitallibrary.un.org/record/4062933, accessed on 25 July 2023), the United Nations Secretary-General highlighted the importance of addressing the digital divide and inequalities related to technology diffusion. These factors affect people’s access to the benefits of technological innovation and risk, further exacerbating social inequalities. For this reason, developing countries should strengthen and enhance their research and innovation ecosystems, providing local actors with the necessary knowledge resources and promoting a favourable institutional and regulatory environment. According to the same source, the digital divide between developed and developing countries is widening in relation to the spread of innovative technologies, with the consequence of relegating the less digitally developed countries to the exclusion of the Fourth Industrial Revolution. However, while developed countries are phasing out old generation networks to adopt advanced networks such as 5G, low-income countries must work with 2G and 3G networks due to obstacles to 5G deployment, including high infrastructure costs, unreliable electricity, and regulatory constraints. It was estimated that only 36% of the population in Least Developed Countries (LDCs) use the internet, compared to the world average of 66%.

Information and communication technologies (ICTs) have yielded benefits in education, health care, and financial inclusion, among others, but their use has been limited for developing countries due to a lack of infrastructure, IT capacity, human resources, and necessary skills. Digital progress around the world can be measured with the E-Government Development Index (EGDI), an index developed by the United Nations (UN) that assesses the ability of governments to provide public services online in an efficient and accessible manner, based on three main components:

1. Online Service Index (OSI)—measures the quality and availability of online services offered by the government.
2. Telecommunication Infrastructure Index (TII)—assesses the level of development of ICT infrastructures (such as internet access, mobile networks, and broadband).
3. Human Capital Index (HCI)—analyses the level of education and digital literacy of the population.

The EGDI is published every two years by the UN, UN E-Government Survey, and provides an up-to-date analysis of the evolutionary state of digital government monitored around the world, with respect to the implementation process of strategies implemented in 193 states. It is an overview, therefore, of how governments are digitising services and promoting digital inclusion. The thirteenth edition of the E-Government Survey report (https://publicadministration.un.org/egovkb/en-us/Reports/UN-E-Government-Survey-2024, accessed on 18 June 2025) was published in 2024.

Countries with extremely high EGDI values, above 0.75 (on a scale of 0 to 1), make up the largest share, representing 39% of the countries assessed. This group is followed by those with high EGDI values, ranging from 0.50 to 0.75, which make up 32%. The number of countries with average EGDI values, ranging from 0.25 to 0.50, decreased to 23%, while countries with low values increased to 6% due to geopolitical conflicts.

Overall, countries with an extremely high EGDI mostly coincide with advanced economies. Central Africa and the Horn of Africa show the greatest difficulties in terms of digitalisation. The digital divide between the Global North and South remains evident, although it is improving in some emerging areas such as India and South America. Over the last 10 years, the number of countries with extremely high EGDI values tripled, from 25 in 2014 to 76 in 2024. More generally, the sum of countries with extremely high and high EGDI values increased from 87 to 138, underlining the commitment made by governments to improve e-government services and infrastructures and the importance attributed to digital transformation. In the face of these increases, the number of countries with medium and low EGDI values has decreased. Comparing the global average EGDI in 2024 with the scores of individual countries, Europe records the highest regional average (0.85), followed by Asia and the Americas. In terms of growth, Asia experienced the highest percentage increase in its EGDI value between 2022 and 2024 (+7.7%), followed by Africa (+4.8%), the Americas, and Oceania (both +4.1%), while Europe showed the smallest growth (+2.3%).

Digital innovative technologies are increasingly adopted to revolutionise safety training, industrial work processes, and the management of occupational safety and health in high-risk industries [5,6,7]. The most significant advantages of VR experiments are practical in nature and related to their perfect adaptation to their purposes. Costs are no longer an obstacle to technological integration, and it is understandable that VR will become increasingly accessible in the near future, partly as a result of updates to standards relating to health and safety training in the workplace and the new State-Regions Agreement in Italy, which regulates training by encouraging the use of VR as an aid in worker training courses [7].

In Italy, occupational safety and prevention training is regulated by Legislative Decree No. 81/2008 of 9 April 2008. Article 37 of the decree, “Training of workers and their representatives”, paragraph 1, states, “The employer shall ensure that each worker receives sufficient and appropriate training in health and safety” [8]. The duration, minimum content and modalities of the training are indicated by the Agreement of the Italian Permanent Conference of the State, Regions and Autonomous Provinces, which has been recently updated [9]. The Agreement, about active teaching methods, makes explicit reference to the opportunities and tools offered by ICT. Examples are augmented reality (AR) and VR, virtual and physical/machine simulators, and gamification. However, the use of VR or AR does not replace the practical part related to the course for workers, employers and self-employed persons working in a suspected polluted or confined environment pursuant to Presidential Decree No. 177/2011 [10] or the practical part for the qualification of operators in the use of hazardous equipment. Moreover, the new agreement introduces significant innovations regarding the evaluation of training effectiveness, making it an integral part of the training process, with the aim of verifying and measuring the actual impact that the training has had on participants.

### 1.2. The Virtual Reality

With the belief that “learning by virtual doing” is more effective than traditional methods, as well as in terms of training effectiveness and safety, lower costs, and motivation of learners through active participation, XR has been widely applied in high-risk industries such as manufacturing, construction, mining, transport, power distribution/thermal plants, aviation, and firefighting [11].

Extended Reality (XR) technologies enrich human experiences by creating immersive computer-generated environments and include VR, AR, and Mixed Reality (MR). VR creates fully immersive virtual worlds that simulate the real one, using electronic devices like sensor-equipped helmets and screens by which users can navigate, interact, and receive feedback based on their manipulations of virtual objects, and AR overlays virtual elements onto the real world, while MR enables real-time interaction between physical and virtual environments, where virtual objects coexist and interact with real-world objects, merging virtuality and reality (OSHA 2025) (OSHA, 2025: Augmented and virtual reality for remote work: Are they safe for workers? Available at https://healthy-workplaces.osha.europa.eu/en/media-centre/news/augmented-and-virtual-reality-remote-work-are-they-safe-workers, accessed on 18 June 2025).

Technically, VR is a computer-generated setting in which individuals act in a real-time simulated environment, creating artificial locations through an interface that stimulates one or more senses. The digitally generated space is such that users’ movements are tracked, and environs are displayed in synchrony with users’ actions.

VR technology can be applied to two types of environments that are differentiated by the degree of users’ immersion. The first types are low-immersive virtual environments (LIVEs), which are computer-screen-based renderings of real environments or virtual worlds, such as Second Life, World of Warcraft, EverQuest, and The Sims, in which users interact with digital models called avatars embodying their virtual selves. The second type is high-immersive virtual environments (HIVEs), which employ specialised displays such as the Cave Automatic Virtual Environment, which comprises enclosed boxes showing images projected on multiple interior screens; head-mounted displays, such as Oculus Rift, Samsung Gear VR or Google Cardboard; or augmented or Mixed Reality devices, like Microsoft Holographic and HoloLens headsets. In these settings, users’ senses are dominated by the technical equipment to a degree related to the adoption of devices, such as headphones, body trackers, gloves, or touch controllers; the extent of the field of view, the quality of rendering and the speed of the interaction with virtual domains [7].

The following subsections provide a review of recent studies regarding the integration of VR technologies into occupational health and safety training programs in various industrial fields.

#### 1.2.1. Application of Virtual Reality in the Manufacturing Sector

Immersive virtual reality training in manufacturing is strongly supported by scientific evidence, and empirical data demonstrate its effectiveness, especially in terms of technical skills, safety, assembly, maintenance training, quality control, new product development, and design evaluation. This technology can improve efficiency, anticipate equipment failures, and optimise operations by providing information through real-time simulations and predictive models [12]. A recent paper reviewed 78 studies to assess whether immersive VR is effective for industrial skills training in industrial areas such as manufacturing, construction, aerospace, and transportation using metrics as NASA-TLX (task load index) for cognitive load, SUS for usability, and SSQ (simulator sickness questionnaire) for simulator sickness [13]. In some cases, traditional physical training outperformed VR, especially when it lacked realistic haptic feedback or when it induced a high cognitive load. The study identified limitations related to focalisation only on performance metrics, ignoring psychological factors like motivation or self-regulation, or limited physical immersion that often reduce realism and learning effectiveness [14]. Both controlled studies and large-scale industry implementations show that VR applications in manufacturing training have become an efficient and effective method for teaching skills and processes to employees, with higher learning effectiveness, efficiency, and knowledge retention by the users. The state of the art, benefits and challenges related to the use of VR in manufacturing training were addressed by Ipsita (2025) [15]. VR training helped learners improve accuracy, retention, and adherence to safety protocols in high-risk tasks; it was shown to be particularly effective in welding, machining, robotics, and additive manufacturing contexts, reducing the cost and exposure to hazards.

Training industrial manufacturing personnel traditionally involves a gap between classroom theory and real-world practice. This gap becomes more pronounced in increasingly complex, technology-driven environments. Li et al. (2025) showed in an empirical study with quantitative data that in this area, the VR platform significantly improves knowledge retention, task execution accuracy, and training effectiveness, thanks to high-fidelity virtual environments, dynamic scenarios, and adaptive feedback [16].

#### 1.2.2. Application of VR in Construction

The construction industry is widely recognised for its high accident rates and hazardous working conditions. In 2020, the sector recorded 4764 fatalities in the United States alone [17]. In 2022, in the EU, 3286 fatal workplace accidents were recorded, of which 22.9% occurred in the construction sector (Eurostat, 2024) [18]. In this area, the use of VR technologies for training purposes is essential, bridging the gap between theoretical knowledge and direct practical experience to improve safety. In this context, novice participants exhibit greater gains from VR training, while experienced workers show smaller learning increases, often reporting higher self-efficacy and trust in the system. The use of VR technologies allows construction workers to efficiently practice and respond to various dangerous scenarios in a controlled virtual environment, helping to reduce the risk of accidents and injuries and reducing training costs [19]. In the construction sector, Nykanen et al. explored the use of VR technology for safety training and concluded that it has the potential to improve safety competence and motivate behavioural changes [20]. A systematic review and meta-analysis covering research from approximately the past decade (2013–2023) showed that VR is significantly more effective in construction safety training than traditional methods for behaviours, skills, and experience, providing robust quantification of VR’s advantages and emphasising the importance of realistic training context and scenarios tailored to job roles for optimal effectiveness [21].

In a recent study [22], the adoption of interactive VR technologies to provide construction workers with immersive training experiences in the critical domain of fall safety has been analysed versus traditional lecture-based training. All training methods were performed with eighty-two workers. Results showed that VR-trained participants made significantly fewer errors post-training, with statistical significance (*p* = 0.0016), with some limitations related to no behavioural or long-term retention assessment and to the sample representation.

A study analysed a selection of seventeen studies for meta-analysis, quantitatively evaluating the impact of AR/VR on learning outcomes and training efficiency in the architecture, engineering, and construction (AEC) industry. Quantifiable positive impacts were represented by improved knowledge retention, spatial comprehension, procedural efficiency and learner engagement, while challenges and limitations of this technology were identified in high hardware and software costs and failed integration issues because AR/VR is not yet seamlessly embedded into workplace training programs and needs more scalability and accessibility and the adoption of uniform metrics to allow broader comparisons and meta-analyses [23].

#### 1.2.3. Application of VR in Healthcare

VR is transforming healthcare, especially in training, therapy, and surgical preparation. It offers measurable improvements in clinical skill acquisition, patient engagement, self-confidence, treatment outcomes, decision-making, and learner engagement, particularly for novice health professionals. Orthopaedic and surgical training shows particularly large benefits, improving performance accuracy [24,25], with limitations due to simulator types, training protocols, and outcome measures heterogeneous, while nursing and emergency simulations offer measurable gains, especially in high-stakes neonatal emergency training [26], with moderate positive effects on knowledge, confidence, and student satisfaction [27].

Its adoption is still tempered by technical costs and evidence-quality challenges. Development and deployment require interdisciplinary teams (software engineers, clinicians, educators), high-performance computing, secure data systems, and ongoing technical support [28].

Moreover, broader deployment and robust multi-site trials with longitudinal follow-up are still needed to confirm durability and real-world impact. Future directions in this framework regard artificial-intelligence-enhanced VR with dynamic patient simulations; intelligent feedback; adaptive scenarios; haptic feedback evolution with better realism in surgical, tactile, or physical therapy applications; and use of MR for overlaying patient data during real procedures [29,30].

#### 1.2.4. Other Applications of VR in Agriculture, ICTs, and Education

Other VR application areas are in agriculture, ICTs, and education. Agriculture is one of the most hazardous occupations in the USA; farming-related injuries and deaths occur, especially involving tractors. The VR intervention enhanced the perceived threat of tractor-related accidents, which in turn led to improved behavioural intentions for safety [31].

Other VR applications regard the use of innovative solutions for farm planning, precision agriculture, remote monitoring, and management. A study highlights the use of VR to simulate real-life farm scenarios, enabling farmers to practice techniques in a safe environment. This approach improves experiential competencies and supports informed decision-making [32].

The advancement of VR technology has garnered increasing attention in ICT, particularly due to the innovative possibilities enabled by fifth-generation (5G) mobile communications. VR in ICT is evolving toward immersive collaboration, metaverse-enabled learning, and intelligent networking platforms, which are particularly useful in the education area to enrich communication and improve user engagement and learning outcomes, particularly in the presence of learning disabilities, also raising important questions about usability, technology readiness, and sustained values [33].

VR in education refers to the use of immersive, computer-generated environments to enhance learning experiences. A recent paper investigated how AR can be integrated into Makerspace Museums to enhance civic education, particularly by promoting multiperspectivism and encouraging critical thinking, empathy, and civic competence. The AR system featured interactive simulations of civic conflicts, role-playing exercises, virtual field trip experiences simulating complex civic scenarios, and collaborative tasks to negotiate or problem-solve civic issues in groups. Students tested the prototype and provided design feedback [34].

VR can simulate social interactions for training in leadership development, empathy training (e.g., simulating the experiences of marginalised groups), shared virtual classrooms, etc. A recent systematic review [35] on VR and gamification in education, covering 112 articles, found benefits among students/teachers regarding increased motivation, engagement, and immersion; better knowledge acquisition; enhanced self-efficacy and collaboration; and personalised learning. Extended use of VR can lead to physical discomfort, including symptoms of “cybersickness” such as nausea and eye strain [36]. These issues are particularly concerning for younger students and can hinder the effectiveness of VR as an educational tool. Current VR technologies may not be fully accessible to students with disabilities [37]. A study highlighted various barriers, including physical, cognitive, visual, and auditory impairments, which can limit the inclusivity of VR-based learning experiences. Moreover, the initial excitement surrounding VR can lead to the “novelty effect”, where students’ engagement diminishes over time as the technology becomes less new and more routine. This can impact the long-term effectiveness of VR in education [38].

The integration of XR into occupational settings raises safety, physical, psychological, cognitive, and privacy-related concerns. For example, AR may distract workers; poorly designed AR interfaces can cause misinterpretation of real-world elements, for instance, misjudging a vehicle’s speed or reaction time; and prolonged use of AR devices can lead to physical discomfort, with eye strain being one of the most reported issues due to close screen proximity. Additional concerns include disrupted circadian rhythms; sleep disorders caused by device illumination settings, cybersickness, and nausea; headache; uncomfortable stomach; numbness; etc. AR use may also pose mental health risks such as stress, anxiety, and depressive symptoms and could alter users’ perception of the physical world, which can influence cognitive functions such as memory and decision-making. Moreover, AR devices pose substantial privacy risks due to their ability to continuously collect visual and sensory data [39] (OSHA, 2025) (Worker exposure to virtual and augmented reality and metaverse technologies: how much do we know? Osha; World Day for Safety and Health at Work 2025: Global Report).

Dodoo et al. identified challenges associated with the use of XR systems in high-risk industries, stemming from both human and socio-technical factors. Further challenges include limited viewing fields, poor signal, and loud noise, which affect users’ VR experience, making it difficult to hear, wear, and walk while using it [5,40]. As for social factors, users noted that the VR system architecture restricted their movements, thereby reducing immersion [40].

Other studies highlight the need for a more standardised approach in the evaluation of the effectiveness of VR for the purpose of occupational health and safety training to ensure more reliable and comparable results across different sectors [41].

As noted by Sabzevari and Dehghan, training plays a critical role in reducing workplace accidents and improving safety. Their 2018 study compared in-person and VR training on personal protective equipment (PPE) usage among seventy-five workers in an open stone quarry. Statistical analyses, including the Chi-square test, paired sample *t*-test, and ANOVA, showed no significant differences in PPE usage between the groups before training, but significant improvements were observed after the sessions [42].

A 2023 review found limited evidence supporting the effectiveness of VR in training applications. The study categorises evaluation metrics according to the four hierarchical levels of Kirkpatrick’s model for training evaluation: level 1, Reaction, measures participants’ immediate responses and attitudes toward the training experience; level 2, Learning, assesses the knowledge and skills acquired during the training; level 3, behaviour, evaluates changes in job-related behaviour following the training; and level 4, results, focuses on concrete outcomes, such as reduced safety incidents, increased productivity, or cost savings. The results showed that the majority of 136 studies evaluated learning and reaction levels, while very few studies evaluated behaviour and results levels. Few studies analysed the impact of VR on actual worker behaviour and concrete outcomes, such as the reduction of occupational accidents [1].

Renganayagalu et al. (2021) [43] highlight critical issues related to reviews investigating the effectiveness of VR training, particularly related to the need for greater standardisation of criteria for evaluating the effectiveness of VR in OSH (Occupational Health and Safety). While VR has been widely used in the healthcare sector to train surgeons, doctors, and nurses and its effectiveness has been validated in other sectors, such as construction, mining, agriculture, and transport, VR training tends to be experimental and lacks assessments of effectiveness or widespread implementation [1,43].

Pedram et al. explored the technological aspects of VR and user experiences, highlighting the importance of factors such as user attitudes towards technology, industry culture, and the adaptability of VR to specific training scenarios [44].

According to Nanadrekar et al., the goal of training is to enable operators to perform their tasks in a manner that meets both company standards and safety requirements. The authors examined factors that contribute to the human efficiency of Dumper Operators in quarries, developing an Operator Skills Matrix (OSM) to guide retraining efforts when needed. Operators play a key role in improving safety and productivity, thereby reducing mining costs (ML3). VR Training is one technique that can support this goal [45]. According to Dodoo et al., safety training is a critical driver for promoting workplace safety. These observations indicate a gap in understanding how XR (e.g., VR, AR, and MR) might contribute to worker safety within high-risk industries through safety training. To address this gap, the authors explored the application of XR technologies in safety training for high-risk industries and identified challenges impacting the effectiveness of XR-based safety training [5]. Slower response times due to cloud rendering hardware were identified as another limitation hindering workers’ adoption of VR technology [46]. Stefan et al. found that in industries like construction, mining, agriculture, and transportation, VR training tends to be experimental and lacks efficacy assessments or widespread implementation [1].

### 1.3. Psychological Impacts of VR in Safety Training for High-Risk Jobs

Safety training is a crucial component in high-risk work environments such as quarries, chemical plants, or complex industrial settings. In recent years, VR has emerged as an innovative tool capable of radically transforming training methods by providing immersive environments and highly realistic simulations. However, while VR presents significant advantages, it is not psychologically neutral and can generate both benefits and critical effects, especially when not tailored to the cognitive and emotional characteristics of the user.

One of the primary psychological benefits of VR in safety training is the enhancement of risk awareness. The ability to recreate hazardous scenarios in a controlled environment allows VR to stimulate experiential memory and improve workers’ ability to recognise danger signals in real-life contexts [47]. This approach is especially useful for less experienced workers, as it enables them to face complex situations without real exposure to danger, thereby boosting their self-efficacy and reducing anxiety [48]. VR’s immersive quality encourages deeper cognitive and emotional engagement compared to traditional methods, increasing attention, motivation, and information retention [49]. The opportunity to repeatedly experience high-pressure simulations also helps develop effective stress management and rapid coping strategies, which are critical during emergencies. In contexts like fire evacuation, VR has proven effective in developing readiness and effective responses [50]. The gamification of VR training can increase engagement and motivation but must be carefully balanced with reflective practices (such as debriefings) to ensure that the seriousness of the content is not diminished.

Despite its benefits, VR use also presents psychological risks that must not be overlooked. One of the most common is cybersickness, a condition characterised by nausea, disorientation, and cognitive fatigue, often caused by overly intense simulations or poorly optimised technology [51]. These symptoms can reduce training effectiveness and negatively impact user well-being. Excessive exposure to simulated danger may also lead to desensitisation, weakening a person’s perception of real-world risk. For individuals who have experienced past trauma, immersive simulations might trigger emotional reactivation or anticipatory stress, making psychological screening and care essential [52]. From an operational standpoint, the effectiveness of VR largely depends on the quality of the technology used, which may be costly or prone to malfunction [53]. Moreover, to maximise learning outcomes, VR should be integrated with traditional methods, such as field exercises, mentoring, or group discussions, to ensure skill transfer to real-life situations.

### 1.4. Study Hypotheses and Research Questions

This work addresses the complex issue of VR training assessment through an analysis of socioeconomic, technological, and regulatory aspects that directly or indirectly influence the training life cycle (TLC). The reference for the study is represented by the training evaluation models available in the literature and the metrics applied in the evaluation of software and hardware quality.

The study aims to answer the following research questions:

1. Are there models to evaluate the effectiveness of workplace health and safety courses based on virtual reality?
2. Are there production sectors where the application of such models could be more critical?

The answer to these questions comes from a systematic analysis involving the TLC and their respective levels, metrics, or requirements that intervene in the evaluation process.

Section 2, includes the study design; outlines the global technology gap, strengths and weaknesses, and psychological impact of virtual reality in health and safety; and includes a review of TEMs and TMs available in the literature. Section 3, presents the new macro-level model for evaluating health and safety training in virtual reality. Section 4 includes a discussion of this work and the limitations of the study. Section 5 presents the conclusions of the work.

## 2. Materials and Methods

### 2.1. Study Design

The study addresses the conceptual development of an integrated model with the first results of empirical studies, including those conducted by Inail on virtual reality as a training tool for health and safety at work [41,54].

Preliminarily, a bibliographic review of the training evaluation models (TEMs) available in the literature is proposed, from Kirkpatrick’s model with its evolutions and integrations to the most recent models [55]. The life cycle of training activities is divided into three macro-levels (MLs), which include the design, delivery, and evaluation stages. Thereafter, the levels of each training assessment model are aggregated at the TLC stages (Table 1). This aggregation on macro-levels integrated with the related metrics is applied to the training life cycle using virtual reality to generate the evaluation model (VR-TEM) [56,57].

### 2.2. VR Training Evaluating Models

The first research question is focused on the existence of evaluation models of health and safety training using VR.

Some evaluation models in the literature focus on design requirements and thus on “demand” for training prior to its implementation. Others evaluate performance during user–training interaction. Yet others instead focus on the experience following the delivery of courses, measuring the effects of training in the short and long term for the subjects covered by the training, for their companies, and for society. Most evaluation models focus on the project phase, which includes the analysis of “needs” or demand for training, the definition and evaluation of the objectives to be achieved, the project phases, and the evaluation of the costs of implementation, acquisition, organisation, and preparation of training courses. Health and safety requirements at work according to Italian legislation are reformulated when new production processes take place, when new regulations come into force, or in the event of accidents, where it is necessary to intervene in the definition of activities and therefore in the consolidation of new student behaviours to replace inadequate behaviours. Some models focus only on intermediate levels where the student’s reactions, the trainer’s performance, and the technologies used in training are evaluated. In the final levels, however, the “fidelity” or short- and long-term effects of the training are evaluated, as well as the return on performance, such as process optimisation, the improvement of operating, and safety conditions, but also the level of satisfaction, involvement, and well-being perceived during and after the course. In many of these cases, feedback is processed with the help of interviews and suggestions provided by the students.

Kirkpatrick’s model (reaction, learning, behaviour, and outcomes) is the best known and the least recent. It includes four levels: reaction, apprehension, behaviour, and outcomes. The level called “reaction” captures students’ personal reactions to the contents, methods, and methods of training, taking into account students’ feelings during the learning phase. Learning, or the second level, measures the learning outcomes of what students should learn and the added value in terms of knowledge in their field. Behaviour, or Level 3, on the other hand, investigates the reflections of training on actual performance in the workplace and on the application of acquired knowledge and the modification of work-related behaviour. These assessments can be obtained several months after training. The results, or the fourth level, evaluate the tangible benefits for organisations resulting from the implementation of the training program. These are evaluations which can be obtained several months after training on the basis of parameters such as productivity and profitability. The model does not consider the stages preceding the learning experience (Table 1) [58,59].

The CIRO model (context, input, reaction, and outcome) includes four levels: context, input, reaction, and outcome. This model emphasises the analysis of the “before training” phase and thus the examination of the operational situation to determine training needs and objectives. The input consists of information on training methods or techniques that can be used to select the best choice of training intervention, while the reaction concerns the collection of participants’ opinions and suggestions on the training programme. Outcome evaluation examines the training results at the immediate, intermediate, and final levels [60].

Stufflebeam (1983) [61] developed the CIPP model (context, input, process, and product) based on four levels: context, input, process, and product. Context evaluation helps to plan and develop programme objectives. Input evaluation helps to determine the project by examining capacity, resources, and the phases of development of training. Process evaluation is concerned with the implementation of the programme and the provision of feedback on the materials, the lecturer, and the content of the training. Product evaluation refers to the results of the programme in terms of output and outcomes [61].

The Bushnell Model (1990) is better known as IPO (input, process, and output) [62]. The input phase evaluates all elements that may influence the effectiveness of the training, such as the competence of the trainer, training materials, facilities, and equipment used. The process evaluates the trainer and his/her role in the planning, design, development, and provision of the material. The output phase evaluates the impact of the training in the short term and can be traced back to the first three levels of Kirkpatrick’s model (participant reaction, acquired knowledge, and improved job performance) with the addition of a further level consisting of the evaluation of the long-term benefits for the organisation, such as productivity, profitability, and customer satisfaction [62].

Further models, such as Phillips’ ROI (2012) model (reaction, learning behaviour, results, and return on investment), extend Kirkpatrick’s model with a fifth evaluation layer, the so-called return on investment (ROI), which is useful to show the value, in financial terms, of an investment in training [63]. Kraiger, Ford, and Salas (1993) [64] proposed the three domains of training results to further explain changes in learning. Their model is based on cognitive, skill-based, and affective outcomes [58,64]. Their model emphasises how the way training is designed, delivered, and implemented influences the impact and effectiveness of training [55,58]. 

Cannon-Bowers et al. proposed a four-level model that includes learning, learner performance, job performance and outcomes by associating effectiveness with training motivation [58].

The Brinkerhoff Assessment Model includes six levels: 1, needs assessment; 2, goals of training; 3, reaction; 4, learning; 5, behaviour; and 6, results. Levels 4 to 6 follow the Kirkpatrick model. The model is based on a systematic evaluation by measuring all elements of the learning design. The six-stage evaluation starts with a needs assessment and identification of the training objectives. It is a circular model based on the principle that training should produce learning changes whose effectiveness should translate into a benefit for the employee and the organisation [58,65,66].

Molenda’s (1996 and subsequent revisions of 2004) model includes six levels: 1, Activity Accounting, examines the volume of training and the number of participants in the programme; 2, Participant Reactions, measures the learner’s satisfaction with the programme; 3, Participant Learning, measures the extent to which learners apply the knowledge and skills taught during the programme; 4, Learning Transfer, measures the transfer of training and measures the application of skills learned in the workplace; 5, Business Impact, measures the improvement in employee performance and whether and how much the improvement affects profitability; and 6, Social Impact, measures the effect that performance change in the organisation has on society [67,68].

Holton (1996) [69] developed an HRD Evaluation and Research Model for evaluating the effectiveness of training based on human performance improvement that includes training motivation. Holton redefined the three successive levels of Kirkpatrick’s (1959) [58] first model to include learning performance, individual performance and organisational performance, which can be assimilated to Kirkpatrick’s levels 2, 3, and 4. The missing element is the first level, reaction, as it should not be considered a primary outcome of training, as it was believed that favourable reactions and learning were not necessarily correlated [58,69].

A 2024 systematic literature review of 52 publications between 2013 and 2021 on the VR application for safety training emphasises that, unfortunately, only 36% of studies included long-term retention assessments [70].

Evaluating the effectiveness of VR training involves a thorough assessment of key performance indicators (KPIs) aligned with the intended learning outcomes. These KPIs may include knowledge retention, task accuracy, completion time, error rates, and trainee satisfaction. Defining clear KPIs in advance allows organisations to assess the impact of VR training and make informed, data-driven decisions to optimise the learning process. Integrating both quantitative and qualitative assessment methods offers a well-rounded evaluation of VR training effectiveness. Quantitative approaches involve gathering objective data through tests such as a pre-test and an immediate post-test to assess the difference in knowledge between these two assessment simulations, and performance metrics, enabling organisations to measure progress accurately. In contrast, qualitative methods, such as questionnaire surveys, verbal descriptions, in situ performance, and observation by the trainer, represent collection tools to capture trainees’ subjective experiences and perceptions [71].

Cheung et al. [55] adopted the modified Kirkpatrick model to evaluate the effectiveness of surgical training, based on six categories, which include accounting for activities (training objectives/success in organisational change) at Level 0, reaction (satisfaction) at Level 1, learning (acquisition of surgical airway skills) at Level 2, behaviour (post-training change in personal strengths) at Level 3, results (organisational or clinical outcomes) at Level 4, and return on investment or expectation (ROI or ROE) following training and other measures related to quality and safety at Level 5 [55,59]. Within these levels, it is possible to apply a wide variety of metrics that allow training to be evaluated according to objective criteria.

## 3. Results

**Figure 1:**
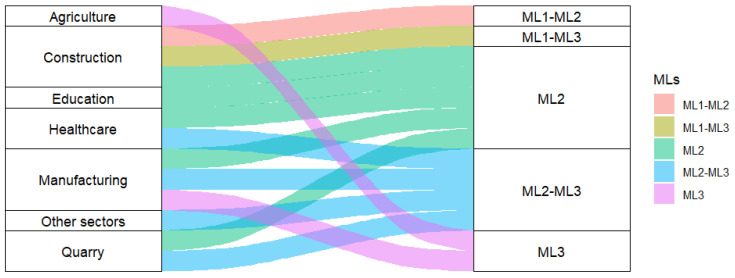
VR-TEM. Occupational sector (first column) vs Macro-Level evaluated (second column). Agriculture: ML3 [32]. Construction: ML1-ML2 [23], ML1-ML3 [19,20], ML2 [22]. Education: ML2 [33,36,37,38]. Healthcare: ML2-ML3 [24], ML2 [25,26,27]. Manufacturing: ML2 [13], ML2-ML3 [14], ML3 [15,16]. Other sectors: ML2-ML3 [45]. Quarry: ML2 [40,41,42], ML2-ML3 [44]. Source: Authors.

To answer the research questions and highlight the gaps in the VR-TEM evaluation models present in the literature, a bibliographic analysis was conducted referring to the different production sectors. The information relating to the sectors involved and the levels being evaluated was traced back to the three macro-levels along the life cycle of virtual reality training and represented on the basis of absolute frequency. The graph in Figure 1 shows that the most frequently evaluated TLC macro-levels are ML2 (Construction, Education, Healthcare, Manufacturing, Quarry), ML2-ML3 (Healthcare, Manufacturing, Quarry, Other sectors), ML3 (Agriculture and Manufacturing), and rarely ML1 in combination with ML2 or ML3 (Construction). The graph highlights the relationships sector-MLs (Figure 1).

As shown in Figure 1, there are no VR evaluation models in the literature that include all three macro-levels involved in the VR-TEM.

### 3.1. TEM and MLs

**Figure 2:**
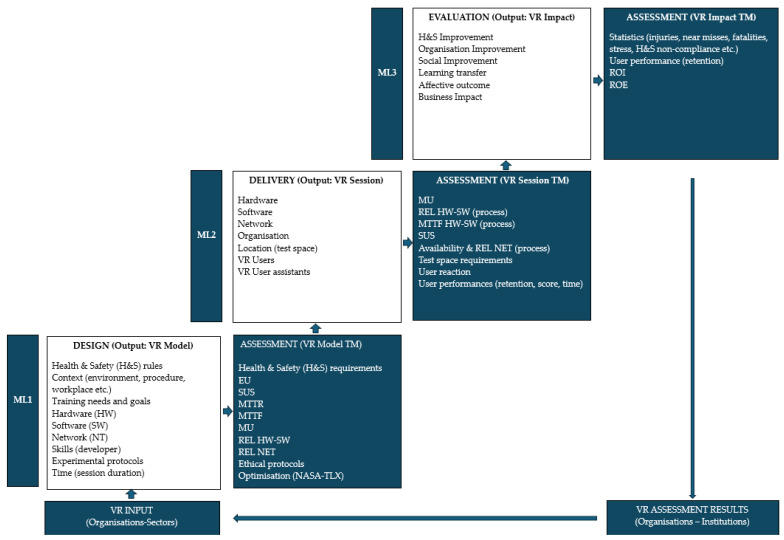
VR-TEM cycle. Development of the model from the VR input to the VR output, along the path defined by the arrows and MLs (Design, Delivery and Evaluation), levels and TMs. Source: authors.

By going through the typical stages of the TLC and drawing on TEMs, such as Kirkpatrick [59] or Molenda [67,68], the study identified the key elements for evaluating the effectiveness of VR training in health and safety. The TEM based on three MLs includes the essential metrics needed to evaluate the effectiveness of VR training throughout its TLC (Table 1 and Figure 2).

As described in Table 1, the proposed TEM is divided into three MLs distributed on the VR-TLC. The new VR-TEM evaluation model in Figure 2 integrates the TEMs described in Table 1, the demand for training, the objectives to be achieved deriving from contractual, and regulatory and contextual requirements for the various organisations and additional requirements relating to ICTs (for example, AI, software, hardware and networks).

In Design (ML1), the suitability of the individual VR experience with respect to working practices is assessed, with adaptation to the scenographic environment and compliance with voluntary and mandatory standards applicable at a local level. According to Kim and Rhiu, in order for a VR system to be accepted, it is necessary to ensure its usability, which is a difficult parameter to evaluate. To this end, the authors developed and proposed a questionnaire to evaluate the usability of virtual reality systems (VRSUQ) using different VR systems and comparing the results of the VRSUQ with those of the System Usability Scale, which is widely used to evaluate perceived usability [72]. In Delivery (ML2), the instructor’s performance, individual reactions, user performance, user psychophysical state, time, and score achieved are evaluated with the aim of monitoring the experience. Finally, in the Evaluation phase (ML3), the subjective outcome of the training or the “modelling” or behavioural change is evaluated, as well as the social and economic impact of the VR training.

The evaluation cycle consists of the definition of the levels included in each individual ML and the application of the relevant metrics that allow you to move on to the next ML. The model in Figure 2 integrates the TEM levels in Table 1, introducing new requirements and parameters relating to ICTs and health and safety requirements in the workplace.

The development of the VR-TEM model represented in Figure 2 takes place according to the path defined by the arrows that connect the sequence of macro-levels, and where progress is allowed after evaluation of the relevant metrics. The cycle proceeds beyond ML3 evaluation with a flow of data and information that converge towards the archives of organisations or institutions (VR Assessment results) responsible for managing statistics (e.g., accidents, near misses and injuries) or defining the need (VR Input) to be applied in specific organisations and production sectors.

In ML1 (Design), organisations or associations from specific production sectors identify the main inputs or resources to be used in the design phase. These resources interact with those provided by developers and contribute to producing the VR TEM. The tangible and intangible resources involved in ML1 are Health and Safety (H&S) regulations, context (environment, procedure, workplace, etc.), training needs and objectives, hardware (HW), software (SW), network (NT), skills (developer), experimental protocols, and time (session duration).

In ML2 (Delivery), organisations or associations from specific production sectors detect learning levels, user performance, and reaction (sickness, satisfaction before, during, after VR training and interview) and processes (the trainer designs, develops, and delivers the program). In ML1, organisations identify the main inputs or resources to be used in the VR training phase. These resources interact with the employees for whom the training is intended and with the staff responsible for conducting the virtual session. The tangible and intangible resources involved in ML2 include, but are not limited to, hardware (headsets, pointers, etc.), software, network, organisation, virtual classroom space, VR users, and VR user assistants.

In ML3 (Evaluation), organisations or associations from specific production sectors identify behaviours (knowledge gained and improved jobs), outcomes (cognitive-skill-based, affective), results, products, outputs, ROI, cognitive improvement, organisational performance learning transfer, business, and social impact.

### 3.2. VR Training Metrics and MLs

The models described can be explained using metrics that represent a mathematical measure of characteristics or properties. Metrics allow us to characterise a wide variety of entities, including the software used in VR training design, by measuring its quality, usability, and adaptability or its performance and short- and long-term impact. By going through the typical stages of the TLC and drawing on TEM models such as Kirkpatrick [59] or Molenda [67,68] and assessment metrics [56,73,74], for instance, usability (SUS), ease of use (EU) [74], reliability (REL), maturity of users (MU) and return on investment (ROI), the model identifies the key elements for evaluating the effectiveness of VR training in health and safety [74,75,76] and describes countless metrics applicable along the TLC and within its MLs (Table 1 and Figure 2).

- SUS is defined as “The effectiveness, efficiency and satisfaction with which specified users achieve specified goals in particular environments” according to ISO 9241-11:2018 (2018) [77]. The effectiveness refers to the ability of a system to achieve its intended goals with accuracy and completeness.
- EU refers to the property of the tool that allows users to produce better results, and in a short time, because of the interactive interface. It makes sure that timely and continual changes are being integrated. The EU metric applied to software, interface, and hardware can be expressed by (i) the learning time (inexperienced users); (ii) the ability to understand (frequent users); (iii) the ability to understand (inexperienced users); (iv) average operational time (frequent users); and (v) average operational time (inexperienced users) [78].
- REL refers to the failure-free operation of any tool. Therefore, the REL of a software testing tool can be evaluated by considering the MTTF (mean time to failure).
- MU refers to (i) the number of years the tool has been used for projects; (ii) the number of customers of the tool using it for more than one year; and (iii) the number of projects using the tool.
- The performance of any tool is hard to measure due to projects of different complexities. Examples of objective performance metrics are efficiency, measured through response times, and effectiveness, measured through error rates.
- ROI refers to the value of corporate training. This metric compares the financial value of the training results with the investment made to achieve those results. ROI is used at the end of the enterprise training evaluation process. It is necessary to compare the time spent studying with the time needed to achieve a positive ROI: if the time spent studying is less, testers will eventually become familiar with the tool, and as a result, only the initial investment will be higher. ROI can be expressed by (i) estimates of safety increase/improvement; (ii) estimates of test timing; (iii) cost per testing hour on average; (iv) estimates of income gain; (v) estimates of implementation cost of the tool; vi. estimates of the increase in quality; (vi) support hours per project; and (vii) the cost per hour of customer support [63].
- MTTR.
- Maintainability Index: This metric provides an overall measure of how maintainable a software component or system is. It combines various factors like complexity, size, and coupling to determine the ease of understanding, maintaining, and evolving the software [56,73,78].

Kim & Rhiu proposed and validated a questionnaire framework (VRSUQ) aimed to assess the VR usability including concepts such as (Un)comfortable, Sense of presence, Complexity, Consistency, Controllability, Compatibility, Disorientation, Vertigo, Enjoyment, Error prevention, Error recovery, Familiarity, Fatigue, Feedback, Frustration, Functionality, Happiness, Ease to use, Motion sickness, Motivation, Naturalness, Nausea, Perceived performance, Preference, Realism, Responsibility, Satisfaction, Seasickness, Comprehensibility, and Willingness to use [72].

#### 3.2.1. ML1 Metrics and Requirements

**Figure 3:**
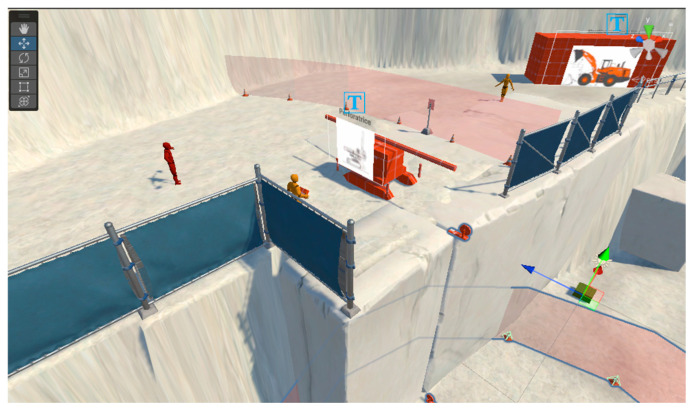
VR TEM. ML1. VR training design on H&S in quarries. Metric and H&S requirement (distances, use of protections, and equipment). Source: Inail DIT and Siena University DISPOC.

Once completed, the VR-based training model is evaluated by means of metrics such as SUS, MTTR, MTTF, MU, Performance, HW REL, SW and NET (project-related) and by means of health and safety requirements (H&S) and applicable ethical protocols (Figure 1, Figure 2 and Figure 3).

During the Design (ML1), in the first exploratory tests of the research project conducted by Inail and the University of Siena (BRiC ID43–2022), the SUS test was applied to 20 volunteers [41]. A questionnaire inspired by the VRSUQ model allowed us to draw the first evaluations on the usability, sickness (Table 2), ease of use, and sense of presence of VR training (Table 3).

#### 3.2.2. ML2 Metrics and Requirements

**Figure 4:**
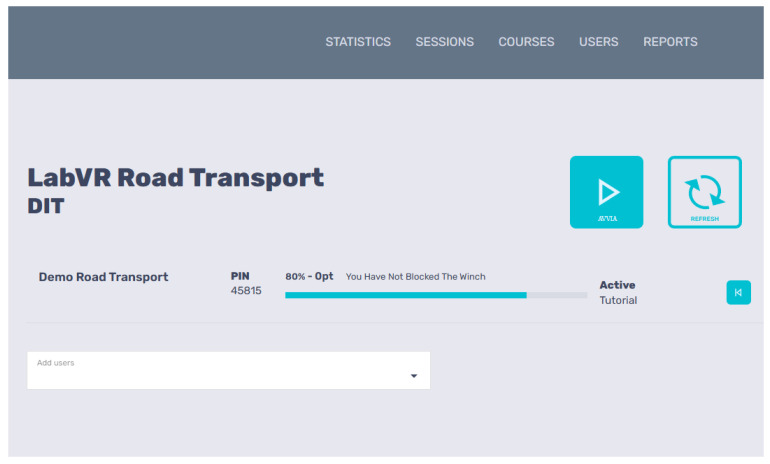
ML2. User performance tracking, including date, duration, percentage of completion, and score obtained in each VR experience. VR training project on health and safety in quarries BRiC ID43-222. Simula Solutions Software v. 1.4.1. Source: Inail DIT and Siena University DISPOC.

**Figure 5:**
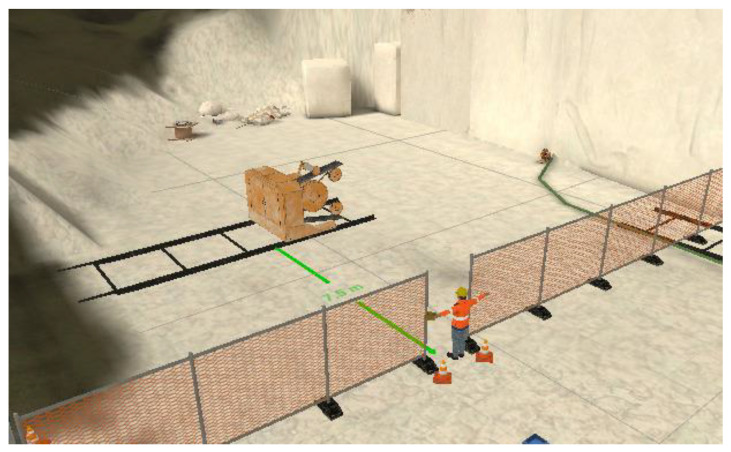
VR TEM. ML1. VR training session on H&S in quarries. VR user interaction on H&S requirements applied to distances, use of protections, and equipment. (BRiC ID43-2022). Source: Inail DIT and Siena University DISPOC.

Once the VR training course has been validated by the developers, the VR model is used and assessed using ITC’s process metrics, such as MU, HW-SW-NET Availability and REL, MTTF, Test space requirements, SUS (user), Reaction, and User performances (retention, score, and time). Finally, the regulatory and ethical requirements involved and the performance and physical reactions of users wearing visors are assessed (Figure 2, Figure 4 and Figure 5).

In the long term, the most common learning objectives are to model or build new behaviours, replace non-compliant ones acquired over time and improve the ability to remember the VR Task and any associated errors after a period following the experience with the headset. The metrics and requirements evaluated in ML3 are based on statistics on injuries, near misses, fatalities, stress, non-compliance, etc.; long-term user performance (loyalty); ROI and ROE (Figure 2); and social and organisational improvement.

## 4. Discussion

VR represents a powerful tool for enhancing safety training in high-risk jobs. Its psychological benefits—such as improved awareness, stress management, and strengthened experiential memory—are substantial but must be accompanied by critical reflection on potential risks. Only through a mindful, human-centred, and evidence-based application can VR effectively contribute to creating safer, more resilient, and more worker-focused environments.

While VR has proven effective for safety training, it also presents several challenges; these include the requirement of high-quality hardware and software, high upfront costs, lengthy development timelines, limited subject customisation, constraints related to both hardware and software, and challenges in creating highly realistic and accurate simulations that mirror real-world scenarios (ML1) [79].

Another crucial challenge is the acceptance of technology among workers. Many studies have examined this issue, employing a range of theoretical models to investigate the factors influencing the adoption or rejection of the technologies. These models are grounded in the Technology Acceptance Model (TAM), which represents a widely used model that explores the acceptance and usage of new e-technology or e-services [80]. It is based on the belief that users’ perception of a technology’s usefulness and EU influences their attitude and intention to use it [14,81]. The TAM model had been used by Rahimi, 2018, to understand the acceptance of clinical staff and patients’ technology adoption [82]. Sagnier, 2020 [83], tested an extended version of TAM with 89 users who performed an aeronautical assembly task in VR. Results suggest that the intention to use VR is positively influenced by perceived usefulness and negatively influenced by cybersickness. Elshafey, 2020 [84], tested by TAM the augmented reality integration in the construction industry, while Haynes, 2024 [85], tested the acceptance of new technologies in the manufacturing area. Another crucial issue is the acceptance and the measurement of the technique’s effectiveness with respect to the age of workers. Pribadi, 2021, showed that VR was an effective and efficient training tool for both graduate students and experienced workers [86]. In this research, acceptance of VR is generally high, and age does not represent a significant factor in learning and using these technologies. While some research suggests that younger workers may be more comfortable with technology, results show that age does not represent a major barrier to learning and using VR and AR [41,43].

Retention measurements (ML3) are particularly important when comparing the effectiveness of VR-based training to traditional methods. The disparity is most evident in long-term outcomes, as shown in studies reviewed by Chittaro and Buttussi and Lovreglio et al. [87,88]. These studies commonly measured retention over periods of 2 to 3 weeks, highlighting a significant limitation in assessing the long-term impact of safety training solutions. The importance of immediate feedback in VR training has been demonstrated by Feng et al. and Burigat and Chittaro. Their findings suggest that immediate feedback is an effective approach for delivering knowledge and enhancing self-efficacy in safety training contexts [89,90]. The VR-TEM significantly integrates psychological dimensions into the TLC, situating itself within the broader context of the Metaverse, a transformative digital ecosystem that merges physical and virtual realms into an immersive, persistent environment. While offering unprecedented opportunities, this convergence also introduces complex ethical and legal challenges, particularly concerning personality rights such as identity, privacy, personal dignity, and emotional well-being (Table 2 and Table 3) [34].

Within ML2, the model explicitly considers individual reactions and the user’s psychophysical state. VR, a core component of the Metaverse, is not psychologically neutral; it can yield both benefits and critical effects if not adapted to the user’s specific cognitive and emotional characteristics [1,43]. The psychological benefits of VR training include enhancing risk awareness by simulating hazardous scenarios, stimulating experiential memory, and improving the ability to recognise danger signals, which is particularly beneficial for less experienced workers [47]. This approach also leads to increased self-efficacy and reduced anxiety, as users can confront complex situations without real danger for workers [45,47,48]. The immersive nature of the Metaverse fosters deeper cognitive and emotional engagement, boosting attention, motivation, and information retention, and helps develop effective stress management and rapid coping strategies through repeated high-pressure simulations [25,31,46,69,90].

However, utilising VR and the Metaverse also presents psychological risks [39]. These include cybersickness, characterised by nausea, disorientation, and cognitive fatigue, often due to overly intense simulations or poorly optimised technology [49]. There is also a risk of desensitisation to simulated dangers from excessive exposure [48,49]. For individuals with pre-existing trauma, immersive simulations might trigger emotional reactivation or anticipatory stress, necessitating psychological screening and support. Physical discomforts such as eye strain, headaches, and neck/back pain can arise from prolonged VR headset use [91,92]. Additionally, technophobia—a psychological aversion or fear of advanced technologies—may exacerbate user resistance to VR environments. Studies have shown that individuals with high levels of technophobia are more likely to experience anxiety, a reduced sense of presence, and lower task performance in immersive settings [93]. Technophobic users may disengage cognitively and emotionally, reducing the overall effectiveness of training programs. Moreover, gender, age, and prior exposure to digital tools significantly influence the degree of technophobic response, as demonstrated by Chua et al., suggesting the importance of adaptive onboarding strategies [94]. Despite these concerns, research indicates that VR acceptance is generally high, and age does not represent a significant barrier to learning and using these technologies [95]. Measurement at this level employs both quantitative methods, like pre- and post-tests to assess knowledge differences and performance metrics, and qualitative methods, such as questionnaires, verbal descriptions, and recommended debriefing sessions for emotional processing and reflection [71]. User maturity, based on metrics like the number of years the tool has been used for projects, the number of customers using it for over a year, and the number of projects employing the tool, is also considered.

In Macro-level 3, the objective is to assess the subjective impact of training, or the “shaping” of behaviour, and the creation of new correct behaviours to replace incorrect ones. This includes verifying the training’s effectiveness in modifying work-related behaviour and applying acquired knowledge. Cognitive and affective outcomes are evaluated, including knowledge retention and the perceived levels of satisfaction, engagement, and well-being during and after the course [71]. Within the Metaverse context, where virtual identity and behaviour can vary, significant challenges exist in verifying virtual identities, potentially amplifying psychological impact due to the immersive nature of the environment [96]. The complexity of evaluation in ML3 stems from behavioural changes being influenced by a variety of internal and external factors that are not always easy to isolate, especially in the dynamic and evolving Metaverse ecosystem. What complicates the assessment of the effectiveness of health and safety training is the need for standardisation [43], compounded by other aspects related to differences that cut across geographical areas, production sectors, and types of companies involved (small, medium, and large).

The novel VR-TEM provides relevant information on the MLs and levels applicable throughout the VR-TLC. The implementation of the novel VR-TEM involves a socio-technical system of OSH skills and technical, legislative, ICT, and psycho-didactic skills, as well as statistical skills. The assessment of needs (ML1) involves a constant comparison between WAI (work-as-imaged) and WAD (work-as-done) instances in companies. This means that VR training in health and safety must be monitored within the organisations where it is implemented, in terms of injuries, accidents, and near miss rates over a convenient period. This constitutes the first limitation of the VR assessment model, which affects those organisations that are not supported by an adequate socio-technical apparatus.

A real, experimental, albeit partial, application of the new VR-TEM concerned the project created by Inail in collaboration with the University of Siena (BRiC ID43-2022). This was a concrete application of ML1 and ML2, the related metrics, and regulatory requirements applicable to the extractive sector. The model, limited to processes simulated with VR, respected the metrics, safety standards, and best practices applicable to the mining sector in Italy (Figure 3, Figure 4 and Figure 5 and Table 2 and Table 3). Referring to the experimentally validated protocols, the score and completion percentage achieved by the forty users were measured in real time along ML2, and the results obtained were stored in a repository made available in the Simula Solutions software v. 1.4.1 (Figure 4). Fifteen days after the experience, the ability of the volunteers who participated in the experiment to remember the correct procedure and the errors made in the interaction with the simulated scenario (retention test) was evaluated. The first results of the retention test in the short term (after 15 days) concerned three groups represented by digital natives (20–35 years), quarry experts (36–50 years), and researchers (over 50 years). The former performed well in terms of score and time but showed poor results in the retention test. The experts in the extractive sector (aged between 36 and 50 years) obtained poor performances in terms of time and score but good performances in the retention test. The researchers obtained intermediate results compared to the other groups [41]. These first results, although of limited statistical significance, represent a useful contribution to the assessment of ML1 and ML2.

There are no results on the impact that the VR training course had within the mining company involved in the project, since no parameters such as ROI and ROE and no statistics were evaluated, nor were the results of the loyalty test evaluated in the long term.

It is conceivable that in the absence of standardisation of MLs in VR TEM and without the involvement of organisations and institutions operating in training, the evaluation of ML3 will remain incomplete.

The first research question concerns the existence of models known in the literature to evaluate the effectiveness of workplace health and safety courses based on virtual reality. Interesting results in the literature evaluate virtual reality training by focusing on certain MLs of VR TLC (Figure 1), thus providing a methodological contribution to the evaluation of specific metrics [72]. However, these assessments overlook countless other aspects along the VR-TLC that have an important impact on the SUS (effectiveness, efficiency, and satisfaction) of virtual reality. There are no studies that address the evaluation of VR training according to a systematic and comprehensive approach along the entire TLC. Furthermore, there is a lack of studies based on adequate sample sizes that provide reliable results on the effectiveness of VR Training in the short and long terms [97]. These aspects represent the main weaknesses of the studies analysed in this work.

Therefore, the study proposes an innovative methodology that systematically places each evaluation process in the right ML, identifying the figures and roles responsible for evaluating the levels and their metrics (Figure 2). The second research question aims to verify whether it is always possible to evaluate the effectiveness of OHS-VR courses using standardised TEMs. In sectors where the production process is continuous and repeatable in space and time (e.g., continuous cycle production lines) or in those where internationally validated operational protocols are used (e.g., surgery), VR training and usability assessment are facilitated (Figure 2). In these sectors, it is conceivable that the standardisation process implicitly included in the VR-TLC model is more easily implementable. The research question was aimed at investigating the production sectors in which the application of standardised VR-TEMs could prove more critical. In sectors such as construction, mining, and many others, where the production process is discontinuous in space and time, technological difficulties may arise that hinder the implementation of standardised VR-TEMs. In the case of training carried out on workers employed on temporary construction sites, in the presence of contractors, long-term retention tests are conditioned by the duration of the work and the contract. In similar work contexts, the evaluation of VR training along ML3 is particularly critical, unless the companies receiving the training and effectiveness evaluation resort to external consortia capable of guaranteeing the application of VR-TEM along all three ML.

The National Institute for Insurance against Accidents at Work could implement ML1 and ML2 in its collaborative research activities and analyse statistics on accidents in the workplace. It remains the responsibility of companies to evaluate ML3 and therefore the long-term social and economic impact of VR training. Further developments in the work aim to identify collaborative agreements with companies in the mining or construction sector aimed at implementing and measuring long-term impact (for example, long-term statistics, ROI and ROE) and, consequently, validate the novel VR-TEM.

## 5. Conclusions

This study aims to ascertain the existence of standardised methodologies to assess the effectiveness of VR-based OHS training. The study shows that there is no model available that can be applied to the entire training lifecycle, but there are many models in the literature that evaluate one or two MLs. The study proposes an evaluation model that develops over three MLs along the VR-TLC, which include Design (ML1), Delivery (ML2) and Evaluation (ML3) and the related TMs. The model and metrics need to be further explored and studied from different points of view due to factors that are beyond the control of the programmers, instructors, and employers involved in the TLC; then, there are socio-economic, regulatory, and psychological and cognitive factors, mentioned in Section 1.1, Section 3 and Section 4. The proposed model, albeit with limitations, stems from the need to exhaustively aggregate all the levels included in the TEMs available in the literature and integrate them with the most current ICT metrics and make them suitable for exhaustively representing the complex reality of the workplace and Occupational Health and Safety issues.

The application of VR-TEM and TM along VR-TLC cannot ignore the specificity of the training and learning objectives that companies intend to achieve, both mandatory and voluntary. These aspects can be clarified on a case-by-case basis, depending on the specific context and in accordance with the recommendations contained in the preceding paragraphs. As regards ML1, the evaluation shall consider the level of adaptation of the simulations to the psychological profile and experience level of the worker. Considering ML2, it is essential to train instructors on empathic communication and emotional support strategies during the viewer experience but also to monitor psychophysical responses throughout the training process, including debriefing sessions to promote emotional processing and reflection. Regarding ML3, it is essential to integrate virtual reality with practical exercises and collaborative learning experiences in enterprises.

## Figures and Tables

**Table 1:** TEM, levels, and MLs in the TLC. Source: Authors.

Author, Year	Model Name, Levels	Macro-Level 1 Design	Macro-Level 2 Delivery	Macro-Level 3 Evaluation
Kirkpatrick, 1996 [58,59]	Kirkpatrick’s, 4	-	reaction, learning	behaviour, results
Warr, Bird, and Rackham, 1979 [60]	CIRO, 4	context, input	reaction	outcome
Stufflebeam, 1983 [61]	CIPP, 4	context, input	process	product
Bushnell, 1990 [58,62]	IPO, 3	input	process	output
Phillips, 2012 [63]	Phillips ROI, 5	-	reaction, learning	behaviour, results, ROI
Kraiger, Ford and Salas, 1993 [64]	Kraiger, 3	input	learning	affective outcome
Brinkerhoff, 1987 [58,65]	Brinkerhoff’s, 6	needs assessment, goals of training	reaction, learning	behaviour, results
Tamkin et al., 2022 [58]	Cannon-Bowers, 4	-	learning	ROI
Brinkerhoff & Dressler, 1990 [66]	Success case evaluation	needs assessment	-	cognitive
Molenda, Pershing, and Reigeluth, 1996 and Molenda and Pershing, 2004 [67,68]	Molenda’s, 6	activity accounting	reaction, learning	learning transfer, business, and social impact
Holton, 1996 [69]	Holton’s human, 3	-	learning and individual performance	organisational performance

**Table 2:** ML1, usability design test. Sickness rating scale 1–4 (1 = none–4 = very). VR training project on health and safety in quarries (BRiC ID43-2022). Source: Inail DIT and Siena University DISPOC.

ID	Symptoms
1	General malaise
2	Feeling of fatigue
3	Headache
4	Eye strain
5	Difficulty focusing
6	Increased salivation
7	Sweating
8	Nausea
9	Difficulty concentrating
10	Mental heaviness
11	Blurred vision
12	Dizziness with eyes closed
13	Dizziness with eyes open
14	Vertigo
15	Stomachache
16	Digestive problems

**Table 3:** VR-TEM. ML1. SUS, EU and PQ design test. Assessment SUS and EU scale 1–5 (1 = strongly disagree to 5 = strongly agree). Assessment PQ scale 1–7 (1 = not at all to 7 = completely). VR training project on health and safety in quarries. (BRiC ID43-2022). Source: Inail DIT and Siena University DISPOC.

ID	Usability Questionnaire
SUS 1	I think I would like to use the headset (or virtual reality environments in general) frequently
SUS 2	I found the headset (or virtual reality environment) unnecessarily complex
SUS 3	I found the headset (or virtual reality environment) very easy to use
SUS 4	I think I would need the support of someone who already knows how to use the headset (or virtual reality environment)
SUS 5	I found the various features of the visor (or virtual reality environment) to be well integrated with each other
SUS 6	I found inconsistencies between the various features of the headset (or virtual reality environment)
SUS 7	I believe that most people can learn to use the visor (or virtual reality environment) easily
SUS 8	I found the visor (or virtual reality environment) very difficult to use
SUS 9	I felt comfortable using the visor (or virtual reality environment)
SUS 10	I needed to learn many processes before I could use the visor (or virtual reality environment) to its full potential
PQ 11	How well were you able to identify sounds?
PQ 12	How well were you able to locate sounds?
PQ 13	How well were you able to probe or search the virtual environment using touch?
PQ 14	How convincing was your sense of moving through space within the virtual environment?
PQ 15	How carefully were you able to examine objects?
PQ 16	How well were you able to carefully examine objects from different points of view?
PQ 17	How well were you able to move or manipulate objects in the virtual environment?
PQ 18	How involved were you in the virtual environmental experience?
PQ 19	How much delay did you perceive between your actions and the expected consequences?
PQ 20	How quickly did you adapt to the experience of the virtual environment?
PQ 21	How capable did you feel of moving and interacting with the virtual environment at the end of the experience?
PQ 22	How much did the quality of the visual display interfere with or distract you from performing the assigned tasks or required activities?
PQ 23	How much did the control devices interfere with the performance of assigned tasks or other activities?
PQ 24	How much could you focus on the assigned tasks or required activities rather than on the mechanisms used to perform those tasks or activities?
PQ 25	How fully were your senses involved in this experience?
PQ 26	How easy was it to identify objects through physical interactions such as touching an object, walking on a surface, or bouncing off a wall or object?
PQ 27	Were there moments during the virtual experience when you felt totally focused on the tasks or the environment?
PQ 28	How easily did you adapt to the control devices used to interact with the virtual environment?
